# Disparities in Preventive Oral Health Care and Periodontal Health Among Adults With Diabetes

**DOI:** 10.5888/pcd18.200594

**Published:** 2021-05-13

**Authors:** Yuqing Zhang, Suzanne G. Leveille, Ling Shi, Sarah M. Camhi

**Affiliations:** 1 College of Nursing and Health Sciences, University of Massachusetts Boston, Boston, Massachusetts; 2 Beth Israel Deaconess Medical Center, Boston, Massachusetts; 3 Harvard Medical School, Boston, Massachusetts; 4 College of Arts and Sciences, Kinesiology Department, University of San Francisco, San Francisco, California

## Abstract

**Introduction:**

People with diabetes are more vulnerable to periodontal disease than those without; thus, practicing preventive oral health care is an important part of diabetes self-care. Our objective was to examine disparities in preventive oral health care among US adults with diabetes.

**Methods:**

We performed a secondary analysis of data from the National Health and Nutrition Examination Survey (NHANES) 2011–2016. Periodontal examinations were conducted in adults aged 30 and older. We compared the weighted prevalence of periodontal disease and the practice of preventive oral health care, including practicing dental interproximal cleaning (flossing or using other interproximal cleaning devices) and use of preventive dental services, among people with and without diabetes. Multivariable logistic regressions were performed to examine the relationship between the presence of diabetes, periodontal disease, and preventive oral health care practices.

**Results:**

Weighted prevalence of periodontal disease in the US population was higher among adults with diabetes than those without (58.0% vs 37.6%). This difference persisted after controlling for sociodemographic characteristics and smoking status. People with diabetes were more likely to have periodontal disease (adjusted odds ratio [aOR] 1.39; 95% CI, 1.17–1.65), less likely to practice daily interproximal cleaning (aOR 0.85; 95% CI, 0.75–0.95), and less likely to visit a dentist for preventive care in the past year (aOR 0.86; 95% CI, 0.76–0.96) than people without diabetes.

**Conclusion:**

Adults with diabetes reported suboptimal preventive oral health care behaviors in use of preventive dental services and interproximal dental cleaning than people without diabetes, despite their health disparity related to periodontal disease. Educating people to improve their preventive oral health care is essential for good oral health and diabetes self-management.

**Summary**

**What is already known about this topic?**

Both diabetes and periodontal disease are chronic diseases in which patients’ self-management skills are critical to prevent disease progression.

**What is added by this report?**

Having regular preventive care dental visits and practicing good oral self-care, such as regular interproximal dental cleaning, are essential preventive measures to improve oral health among people with diabetes.

**What are the implications for public health practice?**

US adults with diabetes report suboptimal preventive oral health care practices compared with people without diabetes, thus emphasizing the need for improved preventive oral health care in this at-risk population.

## Introduction

Periodontitis is an inflammatory chronic gum disease characterized by the formation of periodontal pockets, loss of the gingival attachment, and absorption of the alveolar bone underneath the soft tissue (1). In recent decades, periodontal disease has been linked to systemic diseases and conditions, including diabetes (2–4). Persistent chronic periodontium inflammation, specifically periodontitis, induces insulin resistance, thus jeopardizing glycemic control (5). Highly prevalent but preventable chronic gum disease affects 42% of US adults (6). Previous studies estimated that people with diabetes were 2 to 3 times more likely to have periodontal disease than people without diabetes (7,8).

Oral health care has been a low priority in public health services in the US (9). Notably, Medicare and Medicaid as national health programs help reduce health disparities and inequity in the most vulnerable populations. However, dental care is not included in Medicare, and only basic dental services are sometimes covered through Medicaid. Dental care is the most expensive health care in the US (10). People with diabetes use dental services substantially less that those without, because of financial barriers (11). They are also more likely to have lower satisfaction with their oral health (12,13). Nonetheless, few population-based studies have examined disparities in oral health preventive care behaviors. Because people with diabetes are at increased risk for gum disease, dental visits for preventive care and frequent interproximal cleaning (flossing or using other interproximal cleaning devices) are especially important. The purpose of our study was to assess disparities in periodontal disease and use of oral health preventive care among adults with and without diabetes.

## Methods

### Participants

We performed a secondary analysis of data sets from the National Health and Nutrition Examination Survey (NHANES) 2011–2016, which is conducted by the National Center for Health Statistics (NCHS) (14). NHANES uses a complex, multistage probability-sampling frame among the noninstitutionalized US population. All data used in our study were publicly available and were downloaded from the NCHS website. The institutional review board of the University of Massachusetts Boston determined this project was exempt from its oversight.

We included all participants aged 30 years and over who completed both the NHANES home interview and the mobile examination center examination. Only people aged 30 years or older were invited to participate in the dental examination. We excluded pregnant women and people without teeth. Pregnant women were excluded because they are metabolically different from other women and could have gestational diabetes, which was not the target of our study. We excluded toothless people because their oral self-care behaviors were expected to differ from those of people with teeth.

### Variables

Sociodemographic data (age, sex, race, education, income, and health insurance) and smoking status (never-smoked, former smoker, and current smoker) were collected from the interview questionnaire. Classification of diabetes was based on the participant's answer to the question, “Have you ever been told by a doctor or health professional that you have diabetes or sugar diabetes?” We included only type 1 and type 2 diabetes in our definition of diabetes. People who answered, “borderline,” “refused,” or “don't know” and those with missing data were excluded from our analysis. We chose the self-reported diagnosis of diabetes as the criterion to define diabetes versus no diabetes because the central focus of our study was a comparison of the oral health care behaviors of people aware of their diagnosis of diabetes compared with others. People were classified as having periodontal disease if the measurements on their clinical attachment loss and periodontal pocket depth met the criteria of the case definition for population-based surveillance of periodontitis (including mild, moderate, and severe periodontitis) from the Centers for Disease Control and Prevention and the American Academy of Periodontology (15). Dental service use for prevention or treatment in the past year was determined by asking for a yes or no response to the question, “About how long has it been since you last visited a dentist?” Preventive oral health care behaviors were further measured by whether the participant obtained a preventive dental service and the frequency of practicing interproximal cleaning. Participants who obtained preventive dental service were those who reported visiting a dental professional in the past year with the main reason for the visit being regular teeth check-up or cleaning, called back for check-up or cleaning, or for treatment of a condition identified at a previous check-up. Frequency of interproximal cleaning was measured by asking “Aside from brushing your teeth with a toothbrush, in the last 7 days, how many days did you use dental floss or any other device to clean between your teeth?” People were grouped into 3 categories according to their answer: 7 days (everyday), 1 to 6 days per week, and 0 days (never). The 3 categories were selected empirically on the basis of the distribution of the data, and were the same categories that were used in a previous NHANES study (16). An additional consideration in using this grouping was that until 2016, the final year of our NHANES data, daily flossing was recommended in guidelines of the US Department of Agriculture and US Department of Health and Human Services.

### Statistical analysis

We applied NHANES sampling weights to adjust for oversampling of racial/ethnic minority groups, adults aged 65 and older, low-income people, and for nonresponse to the home interview or medical examination in NHANES 2011–2016 (17,18). The analyses accounted for NHANES complex sampling design. Both the primary sampling unit variable and the pseudo-stratum variable were applied to the analysis by using the SAS Surveyfreq syntax procedure (SAS Institute, Inc). Survey-weighted descriptive statistics were used to present sample characteristics, the prevalence of periodontal disease, any dental visit, use of preventive dental services, and interproximal cleaning among adults with or without diabetes. We used χ^2^ tests to compare the prevalence of periodontal disease, preventive dental care, any dental visits, and interproximal cleaning, according to the presence of diabetes. Multivariable and multinomial logistic regression modeling was used to determine the adjusted odds ratios (aORs) for study outcomes according to diabetes status, controlling for age, sex, race, education, income, and health insurance. All our analyses used a hypothesis test with a 2-sided significance level of .05 and were conducted using SAS version 9.4 (SAS Institute, Inc).

## Results

Of the 29,902 participants from the NHANES 2011–2016 waves, we excluded 15,778 younger than 30, 404 with borderline diabetes, 11 with missing information on self-reported diabetes status, 989 without teeth, and 78 pregnant women. Our final analytic sample size was 12,642.

The weighted average age of the study population was 53.2; 52.0% were women, 67.4% were non-Hispanic White, 10.7% were non-Hispanic Black, 16.4% were Hispanic, and 5.5% were non-Hispanic Asian (Table 1). In terms of education, 30.7% of the population had some college, and 34.1% were college graduates. About 15% of the population had an annual family income below $20,000. Most of the population (84%) had health insurance.

**Table 1:** Weighted Sociodemographic and Smoking Characteristics, US Adults Aged 30 or Older With Or Without Diabetes, National Health and Nutrition Examination Survey (NHANES) 2011–2016 (N = 12,642)^a^

Characteristics	Total, N (weighted %)	No Diabetes, n (weighted %)	Diabetes, n (weighted %)	*P *Value^b^
**All**	12,642	10,718 (88.3)	1,924 (11.7)	NA
**Age, y**				
30–39	2,817 (22.6)	2,719 (24.7)	98 (6.1)	<.001
40–49	2,759 (23.6)	2,500 (24.8)	259 (14.4)	
50–59	2,565 (23.3)	2,136 (22.9)	429 (26.5)	
60–69	2,408 (17.1)	1,789 (15.5)	619 (29.4)	
70–79	1,276 (8.6)	913 (7.5)	363 (17.2)	
≥80	817 (4.9)	661 (4.7)	156 (6.4)	
**Sex**				
Male	6,120 (48.0)	5,128 (47.5)	992 (52.0)	.004
Female	6,522 (52.0)	5,590 (52.6)	932 (48.0)	
**Race/ethnicity**				<.001
Non-Hispanic White	4,769 (67.4)	4,210 (68.5)	559 (59.0)	
Non-Hispanic Black	2,750 (10.7)	2,213 (10.1)	537 (15.3)	
Non-Hispanic Asian	1,629 (5.5)	1,427 (5.5)	202 (5.6)	
Hispanic	3,494 (16.4)	2,868 (16.0)	626 (20.1)	
**Education**				
<High school diploma	2,930 (14.9)	2,328 (14.1)	602 (20.8)	<.001
High school graduate/GED or equivalent	2,702 (20.4)	2,267 (19.9)	435 (23.7)	
Some college or AA	3,582 (30.7)	3,052 (30.4)	530 (32.8)	
College graduate or above	3,418 (34.1)	3,063 (35.6)	355 (22.6)	
**Annual income, $**				
<20,000	2,713 (14.8)	2,170 (13.9)	543 (21.6)	<.001
20,000–74,999	5,928 (47.2)	4,491 (46.3)	937 (53.7)	
75,000–99,999	1,074 (10.8)	957 (11.2)	117 (8.3)	
≥100,000	2,211 (27.2)	2,006 (28.7)	205 (16.4)	
**Health insurance**				
Yes	10,166 (84.5)	8,491 (83.8)	1,675 (89.8)	<.001
No	2,460 (15.5)	2,212 (16.2)	248 (10.2)	
**Smoking status**				
Never-smokeR	7,137 (55.2)	6,113 (55.7)	1,024 (51.1)	<.001
Former-smoker	3,144 (26.7)	2,512 (25.6)	632 (34.9)	
Current-smoker	2,347 (18.1)	2,079 (18.6)	268 (14.0)	

Abbreviations: AA, Associate of Arts degree; GED, general educational development; NA, not applicable.

a Sampling weights were applied to generate US population estimates; groups were compared using the χ^2^ test based on unweighted data.

b *P* values calculated by Pearson χ^2^ test.

Weighted prevalence of self-reported diagnosed diabetes was 11.7% in this US population aged 30 or older (Table 1). Weighted prevalence of any dental visits, regardless of prevention or treatment in the past year, was 63.8% (Table 2). Weighted prevalence of use of preventive dental services was 53.7%, and the proportions never practicing interproximal cleaning, practicing it 1 to 6 days per week, or practicing it every day of the week were 27.2%, 38.6%, and 34.3%, respectively (Table 2).

**Table 2:** Weighted Prevalence of Periodontal Disease and Preventive Oral Health Care Behaviors Among US Adults Aged 30 or Older, With and Without Diabetes, National Health and Nutrition Examination Survey (NHANES) 2011–2016 (N =12,642)^a^

Outcomes	Total, N (weighted %)	No Diabetes, n (weighted %)	Diabetes, n (weighted %)	*P *Value
**All**	12,642	10,718 (88.3)	1,924 (11.7)	NA
**Has periodontal disease^b^, n**	6,690	5,798	892	NA
Yes	3,253 (39.7)	2,669 (37.6)	584(58.0)	<.001^c^
No	3,437 (60.3)	3,129 (62.4)	308 (42.0)	
**Used preventive dental service**				
Yes	5,757 (53.7)	4,982 (54.5)	775 (47.6)	<.001^c^
No	6,617 (46.3)	5,514 (45.5)	1,103 (52.4)	
**Used any dental service**				
Yes	7,271 (63.8)	6,206 (64.3)	1,065 (60.2)	.04^c^
No	5,371 (36.2)	4,512 (35.7)	859 (39.8)	
**Did interproximal tooth cleaning**				
Never	3,977 (27.2)	3,289 (26.6)	688 (31.0)	NA
1-6 days	4,283 (38.6)	3,709 (39.1)	574 (35.3)	<.001^d^
Daily	4,366 (34.3)	3,708 (34.3)	658 (33.7)	.006^e^

Abbreviation: NA, not applicable.

a Sampling weights were applied to generate US population estimates; groups were compared by using χ^2^ tests based on unweighted data.

b NHANES 2011–2014, N = 6,690, was used because dental exams were not conducted in NHANES 2015–2016.

c *P* value calculated by Pearson χ^2^ test based on unweighted data.

d *P* value calculated by Pearson χ^2^ test comparing 1–6 days with never performing interproximal cleaning, based on unweighted data.

e *P* value calculated by Pearson χ^2^ test comparing daily with never interproximal cleaning, based on unweighted data.

Compared with people without diabetes, those with diabetes were less likely to have visited a dentist for treatment or preventive care in the past year (64.3% vs 60.2%, *P* < .05) and more likely to have periodontal disease (37.6% vs 58.0%, *P* < .001). In terms of preventive oral health care behaviors, people with diabetes, compared with people without, were less likely to use preventive care dental services (54.5% vs 47.6%). People with diabetes were also more likely than those without to never practice interproximal tooth cleaning (31.0% and 26.6%, respectively, Table 2). In pairwise comparisons to those who never performed interproximal cleaning, greater proportions of people without diabetes engaged in daily interproximal cleaning (*P* = .006) and cleaning 1 to 6 days per week (*P* < .001) than those with diabetes. After controlling for sociodemographic characteristics and smoking status, people with diabetes were 39% more likely to have periodontal disease (aOR 1.39; 95% CI, 1.17–1.65) and were less likely to practice interproximal cleaning every day than people without diabetes (aOR 0.85; 95% CI, 0.75–0.95) (Table 3). Similarly, adjusting for sociodemographic characteristics, people with diabetes were 14% less likely to use preventive dental service than those without diabetes (aOR 0.86; 95% CI, 0.76–0.96).

**Table 3:** Association Between Diabetes Mellitus and Periodontal Disease, Preventive Dental Service Utilization, and Interproximal Cleaning, US Adults Aged 30 and Older, National Health and Nutrition Examination Survey (NHANES) 2011–2016 (N = 12,642)

Predictor	Outcome
**Has diabetes**	**Periodontal disease^a^^,^ b**
No	1[Reference]
Yes	1.39 (1.17–1.65)
**Has diabetes**	**Uses preventive dental services^c^**
No	1 [Reference]
Yes	0.86 (0.76–0.96)
**Has diabetes**	**Practices interproximal cleaning 1–6 days/week^d^**
No	1 [Reference]
Yes	0.93 (0.82–1.05)
**Has diabetes**	**Practices interproximal cleaning every day^d^**
No	1 [Reference]
Yes	0.85 (0.75–0.95)

a Logistic regression model, adjusted for sociodemographic factors (age, sex, education, income, race, health insurance) and smoking status (never smoked, former smoker, current smoker). Values are adjusted odds ratio (95% CI).

b Used NHANES 2011–2014, N = 6,690, because dental exams were not conducted in NHANES 2015–2016.

c Logistic regression model, adjusted for sociodemographic factors (age, sex, education, income, race, health insurance).Values are adjusted odds ratio (95% CI).

d Multinomial logistic regression model adjusted for sociodemographic factors (age, sex, education, income, race, health insurance), compared to category of never interproximal cleaning. Values are relative risk ratio (95% CI).

## Discussion

We found that US adults aged 30 or older with diabetes practiced poorer preventive oral health care than people without diabetes. Attendance at any dental visit, using preventive dental care services, and practicing interproximal cleaning were significantly lower among people with diabetes than people without diabetes. Previous studies showed health disparities in use of dental services, with a lower prevalence of dental visits, regardless of purpose (prevention or treatment), in the past 12 months among US adults with diabetes than those without (57%–66% with diabetes vs 65%–72% without diabetes) (11,19). Our study not only confirms these disparities in overall dental care, but also shows lower prevalence of preventive dental care visits and poorer oral self care practices among US adults with diabetes.

Prevention plays an important role in reducing risk for many chronic diseases, including diabetes and periodontal disease. A previous study that focused on Medicare beneficiaries aged 65 or older reported that preventive dental care visits occurred less often among people with diabetes than those without (20). The onset of diabetes and periodontal diseases can often be traced to early adulthood. Therefore, from a prevention standpoint, it is necessary to understand whether such disparities are present in the general US adult population. In our study, we found modest differences in preventive dental care visits between US adults with and without diabetes. The prevalence was lower than in other developed countries such as the United Kingdom (85%) (21) and Sweden (95%) (22), but still much higher than in developing countries such as Malaysia (17%–33%) (23,24) and India (11%–28%) (25,26). Factors that might account for these differences include the countries' overall economic characteristics, coverage for costs of care, the population's education level, and the perceived importance of dental care in their health care systems. It is not unexpected that, in low-income countries' health systems, care for conditions that are not perceived as life-threatening ranks low in priority compared with care for other diseases. Both the United Kingdome and Sweden are high social welfare countries. Public health care systems in these countries play a major role in dental health, as opposed to the situation in the US where private dental insurance is the major payor for dental care. Many people in the US do not have dental insurance, and even with dental insurance, often have substantial copayments for dental services.

Previous studies have reported that the perceived importance of oral health in the US is lower than that of general physical health, and oral health issues have been overlooked among people with diabetes (21,27,28). Researchers postulated that the inability to identify signs of oral health problems compounded by the perceived lack of dental needs deterred people with diabetes from receiving timely dental treatment (21,24,29). In our study, we found that although people with diabetes were at increased risk for periodontal disease, they were less likely to practice everyday interproximal cleaning and less likely to obtain preventive dental care. Obtaining regular preventive dental care identifies oral health issues early and creates opportunities for oral self-care education, such as proper flossing and toothbrushing. For much of the US, oral health education mainly takes place in the dentist’s office. A broader public health approach regarding oral hygiene could lead to better oral self-care and more timely dental treatment. This is particularly relevant to people with diabetes, because regular dental visits for preventive care would lead to timely treatment of periodontal inflammation and thus may improve glycemic control.

Efforts to include preventive dental services in current health insurance coverage may partially mitigate oral health disparities experienced by those who are uninsured. Several promising policy changes have been proposed at the US federal level. For example, a proposal has been made to add dental coverage under Medicare Part B with a bill passed in the House (HR.3, Elijah E. Cummings, Lower Drug Costs Now Act). Under the Affordable Care Act (ACA), passed in 2010, some states expanded dental coverage with the expansion of their Medicaid population (10). From 2006 through 2012, total dental visits in the US declined by 7%; however, the number of dental visits in hospital emergency departments and federally qualified health centers increased by 20% and 74%, respectively (30). As part of health reform under the ACA, qualified community health centers received more federal funds to expand their services to low-income and uninsured populations. As a result, dental professional associations encouraged dentists to contract with community health centers in an innovative public–private partnership model (30). Nevertheless, the long-term effectiveness of these improvements that ACA brought remain unclear. In addition, with the current uncertainty about the future of ACA, greater efforts are needed to include preventive dental care services in the public health scheme. These efforts will be even more urgently needed after the current COVID-19 pandemic, which has drastically reduced engagement in preventive dental care services (31). Lastly, more research is needed to evaluate the effect of dental insurance policy changes in this field.

Everyday interproximal cleaning is an important oral hygiene technique for maintaining gum health. It helps to control gingivitis, which is the mildest form of periodontal disease, and prevents the progression of periodontitis (29). Our finding, that only one-third of US adults aged 30 or over with diabetes performed interproximal cleaning every day, was similar to previous reports (32–35). An earlier NHANES study from the 2009–2010 wave reported that only one-quarter of US adults with diabetes practiced interproximal cleaning daily (32). Compared with that study, our findings showed an overall improvement in prevalence of interproximal cleaning. The improvement might be explained by the increasing awareness of flossing gained from modern social media or as a result of generally improved oral self-care behaviors in recent years resulting from the many innovations in oral health care products, such as dental floss picks, interdental brushes, and the water flosser. In a previous study that used a subset (NHANES 2011–2014) of our sample (NHANES 2011–2016), Fleming and colleagues reported no difference in practice of daily interproximal cleaning between people with diabetes and those without after controlling for age, sex, race/Hispanic origin, poverty, and education, by performing a multiple logistic regression analysis where daily flossing was compared with nondaily flossing (combined never flossing with flossing 1–6 days/week) (16). In our study, we considered that people who never practiced interproximal cleaning might be fundamentally different in terms of their self-care behaviors from those who practiced 1 or more days of interproximal dental cleaning in a week. Therefore, we separately compared daily and less than daily cleaning to never cleaning, controlling for sociodemographic characteristics and health insurance. We found people with diabetes were 15% less likely to practice daily interproximal cleaning than those without diabetes. Of note, although daily flossing was recommended in guidelines of the US Departments of Agriculture and Health and Human Services until 2016, it remains unclear whether there is any clinically meaningful difference in gum health between flossing multiple days per week versus daily flossing. In a previous NHANES study, there was no dose–response effect of interproximal cleaning on improving periodontal health (36). In our study, the focus was on behavior in oral care among people with diabetes and those without rather than on the clinical efficacy of oral self-care behaviors on periodontal health or glycemic control. These research questions may warrant further prospective studies.

Our study had some limitations. First, not all variables relevant to our study were collected in the NHANES adult population. For example, frequency of tooth brushing was only asked among participants aged 3 to 19 years in NHANES. Thus, the primary measure of oral self-care behavior available for our study was frequency of interproximal tooth cleaning. We used a measure of health insurance as a limited proxy for dental insurance because no measure of dental insurance was available in the data set. Thus, we cannot conclude that dental insurance could have an effect on this disparity in oral health care behaviors of people with diabetes. This would be an important consideration for future studies. The findings of our study highlighted the importance of oral self-care behaviors, but we acknowledge that future studies and national health surveys are needed that use more comprehensive measures of oral self-care behaviors among adults and older adults. Second, the measure of use of preventive dental care might have been underestimated by combining 2 NHANES questions (ie, “About how long has it been since you last visited a dentist?” and “What’s the main reason you last visited the dentist?”). However, we expect this contributed to a conservative bias, and results from the additional analysis of any dental visits in the previous year showed similar differences between groups.

In summary, our study findings show the oral health disparities experienced by US adults with diabetes and underscore the importance of oral self-care and preventive dental care in this population. To address the disparities in oral health among people with diabetes, it is important that all health professionals who see adults with diabetes reinforce the importance of preventive oral health care. Teaching people with diabetes to perform everyday oral self-care, such as tooth brushing and interproximal cleaning, is a key component of diabetes self-management. Interdisciplinary health care teams not limited to dentists and dental hygienists could help in promoting oral self-care and in improving oral health in the care of people with diabetes, especially in this time of reduced preventive care services related to the COVID-19 pandemic. In addition, public health efforts could bring attention to this health risk through public education and policy innovations targeting populations whose financial status influences access to regular preventive dental care.
